# Evaluating the efficacy of rimegepant as a preventive treatment for chronic and episodic migraine: a three-month longitudinal retrospective cohort study

**DOI:** 10.3389/fneur.2026.1776712

**Published:** 2026-06-04

**Authors:** Hamza Khan, Muhammad Saqib Khan, Sheraz Khan, Fang Liu, Quan Feng, Irfan Ahmad, Ge Tan

**Affiliations:** 1 Department of Neurology, The First Affiliated Hospital of Chongqing Medical University, Chongqing, China; 2 Department of Rehabilitation Medicine, The 2nd Affiliated Hospital of Chongqing Medical University, Chongqing, China

**Keywords:** CGRP receptor antagonist, headache, migraine prevention, real-world evidence, rimegepant

## Abstract

**Background:**

Rimegepant, an oral calcitonin gene-related peptide receptor antagonist approved for both acute and preventive migraine treatment, demonstrates efficacy in clinical trials, but real-world evidence remains limited. This study examined the association between rimegepant use and changes in migraine frequency and patient-reported outcomes in a real-world clinical setting.

**Methods:**

This retrospective cohort study conducted at The first affiliated hospital of Chongqing Medical University, analyzed medical records of 40 adults (aged 18–65 years) with chronic (*n* = 21) or episodic migraine (*n* = 19) who received rimegepant 75 mg every other day for 3 months. Primary outcomes were changes in monthly headache days (MHD) and monthly migraine days (MMD) from baseline. Secondary outcomes included: Headache Impact Test-6 (HIT-6), Migraine Disability Assessment (MIDAS), and Migraine-Specific Quality of Life Questionnaire (MSQ).

**Results:**

Forty participants (mean age 41.12 ± 10.86 years; 80% women) were included, comprising 21 with chronic migraine and 19 with episodic migraine. All five outcome measures improved significantly from baseline to Month 3 (all *P* = 0.001). Monthly migraine days decreased from 17.27 ± 8.52 to 2.90 ± 2.91, and monthly headache days from 19.10 ± 7.46 to 5.60 ± 3.81. HIT-6 and MIDAS scores declined markedly (62.27 to 45.47 and 50.99 to 12.44, respectively), while MSQ scores improved from 59.94 to 86.53. Both chronic and episodic migraine subgroups showed significant improvements across all outcomes, with the greatest reductions in headache frequency occurring between baseline and Month 1. By Month 3, outcome values were comparable between subgroups despite substantial baseline differences.

**Conclusions:**

In this retrospective cohort of patients with prior inadequate response to preventive therapy, Every-other-day rimegepant was associated with meaningful reductions in migraine frequency and headache-related disability over 3 months in both chronic and episodic migraine patients. These preliminary findings are encouraging but require confirmation in prospective controlled studies.

## Introduction

Migraine is a debilitating neurological disorder affecting over 1.16 billion people worldwide, with prevalence increasing by 58% between 1990 and 2021 (1–3). It remains the second leading cause of years lived with disability globally, disproportionately affecting women of working age and imposing estimated annual costs of $78 billion in the United States alone (4, 5). The identification of calcitonin gene-related peptide (CGRP) as a central mediator of migraine pathophysiology (6), driving neurogenic inflammation, pain transmission, and vascular changes during attacks (7), has transformed the therapeutic landscape from non-specific repurposed agents to targeted molecular interventions (8).

Traditional migraine preventive therapies, including beta-blockers, antiepileptics, and tricyclic antidepressants, were developed for other indications and repurposed for migraine prevention (9). These medications are associated with considerable adverse effects and poor adherence, with discontinuation rates exceeding 60% within the first 6 months of treatment (10). The limited efficacy and tolerability profile of conventional preventives have created a substantial unmet need for migraine-specific therapies that offer both effectiveness and favorable safety profiles (11). Patients with chronic migraine, defined as experiencing 15 or more headache days per month with at least 8 days meeting migraine criteria (12), face particularly limited options and often experience inadequate symptom control (13).

The development of small-molecule CGRP receptor antagonists, known as “gepants,” represents a paradigm shift in migraine management (14). Unlike monoclonal antibodies targeting the CGRP pathway, gepants offer oral administration, rapid onset of action, and the potential for both acute and preventive treatment (15). Rimegepant, an orally administered CGRP receptor antagonist with high selectivity and bioavailability, emerged as a leading candidate in this therapeutic class (16). The molecule's favorable pharmacokinetic profile, characterized by rapid absorption and sustained receptor occupancy, provides a mechanistic foundation for its dual indication potential (17).

Rimegepant received FDA approval in February 2020 for the acute treatment of migraine, based on pivotal Phase 3 trials demonstrating superior efficacy compared to placebo in achieving pain freedom and freedom from the most bothersome symptom at 2 h post-dose (18). In a historic milestone, rimegepant became the first and only medication approved for both acute and preventive migraine treatment when it received FDA approval for migraine prevention in May 2021 (19). The approval was supported by the BHV3000-201 study, a randomized, double-blind, placebo-controlled trial demonstrating significant reductions in monthly migraine days and excellent long-term safety (20).

However, the regulatory and clinical landscape for rimegepant in China differs from the United States. The National Medical Products Administration (NMPA) granted marketing authorization for rimegepant in January 2024 specifically for the acute treatment of migraine, without an initial indication for prevention (21, 22). Nevertheless, the clinical application is evolving through expert consensus; the Chinese Guidelines for the Diagnosis and Treatment of Migraine explicitly recommend rimegepant for the preventive treatment of episodic migraine (23), though a formal recommendation for chronic migraine prevention remains pending due to limited specific data (24). Consequently, the use of rimegepant for chronic migraine prevention in China currently represents an off-label application grounded in biological plausibility and unmet clinical need (23). Given the shared CGRP-mediated pathophysiology between episodic and chronic migraine (25), and the lack of tolerable oral preventive options in the Chinese healthcare setting (26), evaluating the efficacy of rimegepant in chronic migraine patients is clinically warranted. This study, conducted in Chongqing, seeks to contribute the local real-world evidence necessary to inform future regulatory updates and guideline refinements regarding this specific patient population.

Despite the promising efficacy and safety profile demonstrated in controlled clinical trials, real-world evidence of rimegepant's effectiveness across diverse patient populations and clinical settings remains limited (27, 28). Most published studies have focused on highly selected trial populations with specific inclusion and exclusion criteria that may not reflect the heterogeneity of patients encountered in routine clinical practice (29). Furthermore, comparative data between patients with chronic vs. episodic migraine receiving rimegepant prevention are sparse, despite the distinct clinical profiles and treatment challenges presented by these two migraine subtypes. Understanding the real-world effectiveness of rimegepant, particularly across different migraine phenotypes and using comprehensive patient-reported outcome measures, is essential for informing clinical decision-making and optimizing patient care.

This study aimed to evaluate the real-world effectiveness and tolerability of rimegepant as preventive therapy for migraine in a diverse clinical population over a three-month period. We assessed changes in migraine frequency, pain intensity, and multiple patient-reported outcome measures, with particular attention to differential responses between chronic and episodic migraine patients. By examining both traditional efficacy endpoints and comprehensive quality-of-life measures, this study provides clinically relevant insights into the role of rimegepant in contemporary migraine management and contributes to the growing body of real-world evidence for CGRP-targeted therapies.

## Materials and methods

This single center, longitudinal retrospective cohort study was conducted at department of neurology, the first affiliated hospital of Chongqing Medical University. Data was collected from medical records of patients with migraine who had been prescribed rimegepant as preventive therapy in routine clinical practice. The study protocol was approved by the institutional review boards, [Chongqing first affiliated hospital ethical review committee (No: KX2026-KYC288-01)].

Patients were included if they had: (1) a documented diagnosis of chronic or episodic migraine according to the International Classification of Headache Disorders, 3rd edition (ICHD-3) criteria; (2) migraine with or without aura, with symptoms fulfilling diagnostic criteria for at least 1 year prior to rimegepant initiation; (3) for chronic migraine patients specifically, a history of 15 or more headache days per month over the 3 months preceding treatment, with at least 8 days per month having migraine features; (4) a documented history of inadequate response to standard first-line preventive therapies; inadequate response was defined as, failure to achieve ≥50% reduction in headache frequency after at least 8 weeks at a tolerated dose, or discontinuation due to intolerable side effects (5) completed baseline assessments and the 3-month uses of the drug and follow-up visits by end of each month.

We excluded patients who: (1) were outside the 18 to 65 year age range; (2) were using other migraine preventive medications during the observation period that could confound assessment of rimegepant effectiveness; (3) had participated in another clinical trial within 90 days prior to rimegepant initiation; (4) had documented history of drug or alcohol abuse; (5) had primary or secondary headache disorders other than migraine that could confound diagnosis.

### Treatment protocol

All included patients had received rimegepant 75 mg orally disintegrating tablets administered every other day for a period of 3 months. This dosing regimen was consistent with the FDA-approved preventive indication. Patients attended routine follow-up visits at monthly intervals (month 1, month 2, and month 3) as part of standard clinical care. At each visit, patients completed standardized questionnaires and provided their headache diaries for review by the treating clinician.

### Outcome measures

The primary efficacy outcomes were changes in monthly headache days (MHD) and monthly migraine days (MMD) from baseline through the 3-month observation period. These were assessed using patient-maintained daily headache diaries, which patients were instructed to complete throughout the treatment period. Headache days were defined as any day with a headache lasting at least 4 h or requiring use of acute medication. Migraine days were defined as days meeting ICHD-3 criteria for migraine.

Secondary outcomes included three validated patient-reported outcome measures. The Headache Impact Test (HIT-6) is a six-item questionnaire that assesses the impact of headaches on ability to function at work, school, home, and in social situations, with scores ranging from 36 to 78 and higher scores indicating greater impact. A reduction of 5 or more points represents a clinically meaningful improvement (30). The Migraine Disability Assessment (MIDAS) questionnaire measures headache-related disability across five dimensions over the preceding 3 months, with total scores categorized as grade I (minimal disability, 0–5 points), grade II (mild disability, 6–10 points), grade III (moderate disability, 11–20 points), or grade IV (severe disability, 21 or more points) (31). The Migraine-Specific Quality of Life Questionnaire (MSQ version 2.1) evaluates migraine-specific health-related quality of life across three domains: role function-restrictive (7 items), role function-preventive (4 items), and emotional function (3 items). Scores are transformed to a 0–100 scale, with higher scores indicating better quality of life (32).

We also collected descriptive information on pain characteristics, including pain intensity (categorized as mild, moderate, or severe), pain duration (categorized as 1–12 h, 13–24 h, or more than 24 h), and the effect of physical activity on headache symptoms. Baseline demographic and clinical characteristics were extracted from medical records, including age, sex, migraine diagnosis (chronic vs. episodic), baseline headache frequency, and baseline scores on all outcome measures. Follow-up data were collected from documentation of each monthly visit, including headache diary information and completed questionnaires. All data were de-identified prior to analysis and entered into a secure electronic database.

We reviewed medical records of all patients prescribed rimegepant for migraine prevention at the Department of Neurology, Chongqing First Affiliated Hospital, between April 2024 and September 2025. During this period, total of 50 patients received prescriptions of rimegepant as preventive therapy. Of these, 10 were excluded for the following reasons: lacked complete baseline assessments, had fewer than 3 months of follow-up data, were using concomitant preventive medications, and the final analytical sample comprised 40 patients with complete data across all four time points.

Note: At our department of Neurology, patients initiating new preventive therapies are routinely scheduled for monthly follow-up visits during the first 3 months of treatment as part of standard clinical care. As institutional practice, all migraine patients attending these follow-ups complete the HIT-6, MIDAS, and MSQ questionnaires, and submit their daily headache diaries for clinician review. This is a longstanding clinical protocol at our center, not a procedure implemented specifically for research purposes. The present study is retrospective in that it analyzed clinical data that had been collected as part of this routine care pathway, without any additional study-specific assessments, interventions, or visits beyond what would have occurred in standard practice. No prospective study protocol was registered, no randomization occurred, and treatment decisions were made solely by the treating clinician based on clinical judgment.

Given the retrospective design using de-identified medical record data, the requirement for individual informed consent was waived. Patient confidentiality and data security were maintained throughout the study in compliance with applicable privacy regulations.

Adverse events were identified through retrospective review of clinical notes documented during each scheduled monthly follow-up visit. Clinicians recorded patient-reported symptoms at each visit as part of routine clinical care. However, no formal standardized adverse event reporting instrument (e.g., CTCAE) was used, and systematic severity grading and causality assessment were not performed. Laboratory monitoring, including hepatic function tests, was not part of the routine follow-up protocol for this cohort. This is a recognized limitation of the retrospective design.

### Statistical analysis

The data of up to 40 patients who completed the 3-month study was analyzed. The distribution of all continuous variables was first assessed using the Shapiro–Wilk test. Based on these results, parametric methods were applied to normally distributed data, while non-parametric alternatives were used when normality assumptions were violated. Data was presented as mean (SD) for continuous variables and frequencies and percentage for categorical variables. To evaluate changes across the four assessment time points (baseline, month 1, month 2, and month 3), a repeated-measures ANOVA was employed for normally distributed variables, and the Friedman test was used for non-normally distributed variables. Finally, all statistical analyses were performed using Python 3.12 with SciPy and stats models packages and SPSS-27 (IBM, USA).

## Results

### Baseline characteristics

A total of 40 participants were included in the analysis. The cohort had a mean age of 41.12 ± 10.86 years and a mean weight of 57.20 ± 8.84 kg, with a mean height of 5.17 ± 0.22 feet. Women accounted for the large majority of the sample (32/40, 80%), while men comprised the remaining 20% (8/40) (Table 1).

**Table 1:** Baseline demographic and clinical characteristics of participants (*N* = 40).

Variables		Mean (SD) frequencies (percentages)
Age		41.12 ± 10.86
Height (feet)		5.17 ± 0.22
Weight (kg)		57.20 ± 8.84
Gender (Male/Female)		8/32 (20/80)%
Accompanying symptoms	Nausea	9 (22.5%)
	Photophobia and phonophobia	14 (35%)
	Nausea plus photo and phonophobia	11 (27.5%)
	Nausea plus vomiting	6 (15%)
Duration	1–5 years	11 (27.5%)
	6–10 years	14 (35%)
	11+ years	15 (37.5%)
Headache location	Forehead	18 (45%)
	Left side of head	20 (50%)
	Right side of head	2 (5%)
Nature of Pain	Pulsating pain	20 (50%)
	Throbbing pain	18 (45%)
	Explosive pain	2 (5%)
Relation with activities	Aggravated with activities	32 (80%)
	No effect of activities	8 (20%)
Pain Duration	1–12 hq	8 (20%)
	13–24 h	18 (45%)
	25+ h	14 (35%)

Values are presented as mean ± standard deviation for continuous variables and frequency (percentage) for categorical variables. Pain duration refers to the average duration of a single headache episode as reported by participants.

The accompanying symptom profile was heterogeneous. Combined photophobia and phonophobia was the single most frequent symptom complex, reported by 14 participants (35%), followed by the combination of nausea with photophobia and phonophobia in 11 (27.5%), nausea alone in 9 (22.5%), and nausea with vomiting in 6 (15%). Headache history was longstanding in most participants: 15 (37.5%) reported a duration of 11 years or more, 14 (35%) reported 6–10 years, and 11 (27.5%) reported 1–5 years.

With respect to pain characteristics, headache was most often unilateral on the left side (20/40, 50%) or located in the forehead (18/40, 45%), with right-sided pain reported by only 2 participants (5%). The nature of the pain was most commonly described as pulsating (20/40, 50%) or throbbing (18/40, 45%), and explosive in 2 (5%). Pain was aggravated by routine physical activity in 32 participants (80%). The duration of an individual attack was 13–24 h in 18 participants (45%), 25 h or more in 14 (35%), and 1–12 h in 8 (20%) (Table 1).

### Treatment outcomes in the overall population

All five outcome measures changed significantly between baseline and Month 3 in the overall cohort (all *P* = 0.001; Table 2).

**Table 2:** Overall treatment outcomes across follow-up intervals (*N* = 40).

Outcome	Baseline	Month 1	Month 2	Month 3	*P*-value
MMD (days)	17.27 ± 8.52	8.20 ± 4.96	4.70 ± 3.62	2.90 ± 2.91	0.001^*^
MHD (days)	19.10 ± 7.46	11.95 ± 6.58	7.83 ± 5.02	5.60 ± 3.81	0.001^*^
HIT-6	62.27 ± 5.64	55.55 ± 5.83	48.05 ± 6.95	45.47 ± 7.21	0.001^$^ (0.80)^@^
MIDAS	50.99 ± 19.24	38.18 ± 15.92	26.36 ± 13.86	12.44 ± 10.58	0.001^$^ (0.83)^@^
MSQ	59.94 ± 12.21	75.30 ± 9.60	82.82 ± 11.32	86.53 ± 10.71	0.001^$^ (0.79)^@^

Data are presented as mean ± standard deviation. MHD = monthly headache days; MMD = monthly migraine days; HIT-6 = Headache Impact Test-6; MIDAS = Migraine Disability Assessment; MSQ = Migraine-Specific Quality of Life Questionnaire. ^*^P-values calculated using Friedman test; ^$^*P*-values calculated using repeated measure ANOVA, *P* < 0.05 considered statistically significant, ^@^Partial eta squared (η^2^*p*).

**Figure 1:**
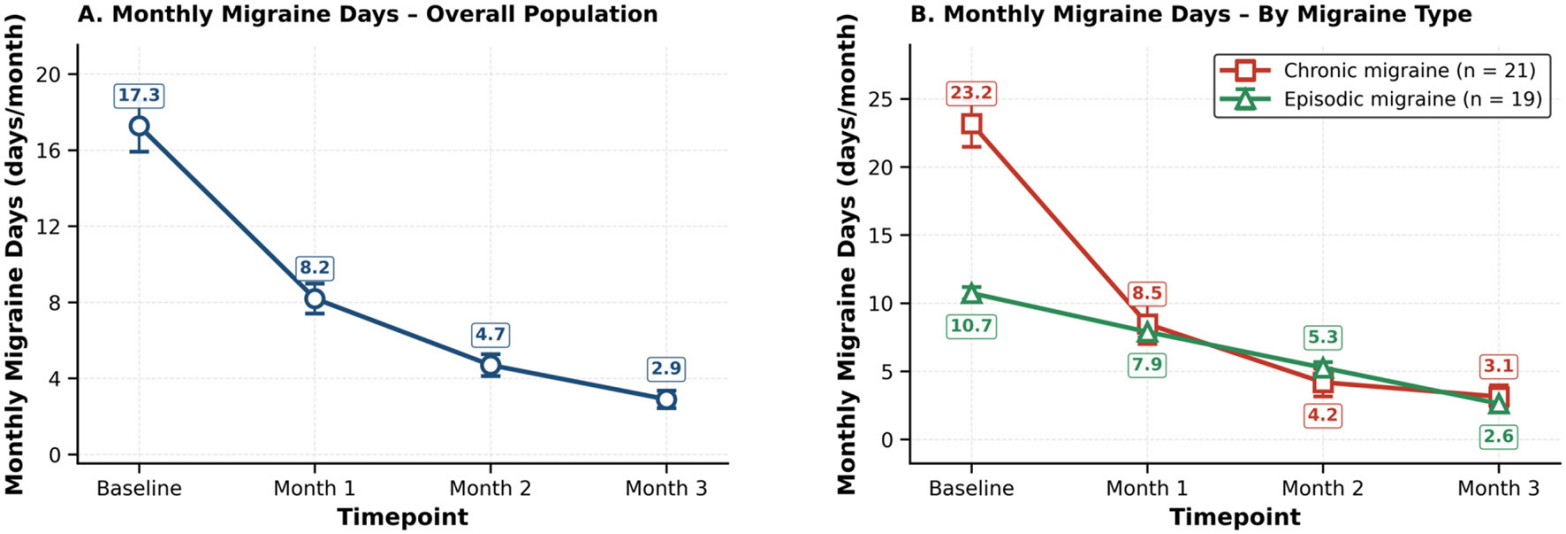
Monthly migraine days. **(A)** Monthly migraine days overall population. **(B)** Monthly migraine days by migraine types.

**Figure 2:**
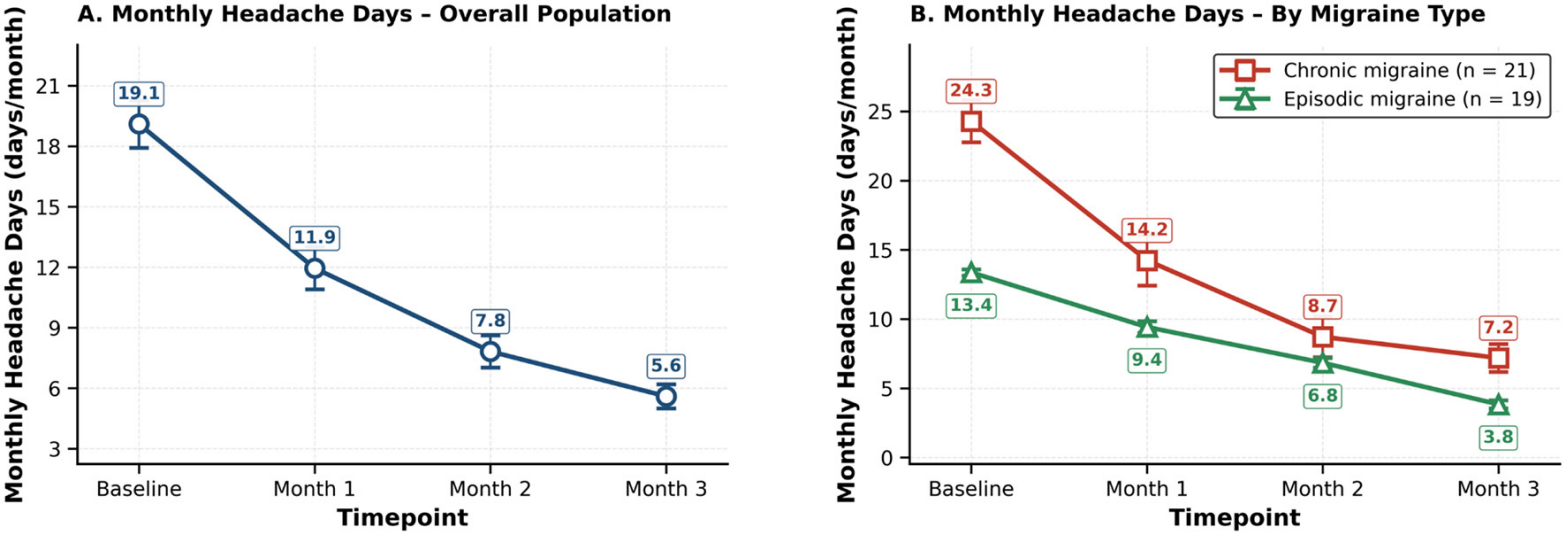
Monthly headache days. **(A)** Monthly headache days-overall population. **(B)** Monthly headache days by migraine type.

Monthly migraine days (MMD) decreased progressively from 17.27 ± 8.52 at baseline to 8.20 ± 4.96 at Month 1, 4.70 ± 3.62 at Month 2, and 2.90 ± 2.91 at Month 3, corresponding to an absolute reduction of 14.37 days (Figure 1A). Monthly headache days (MHD) followed a similar pattern, falling from 19.10 ± 7.46 at baseline to 11.95 ± 6.58, 7.83 ± 5.02, and 5.60 ± 3.81 at Months 1, 2, and 3, respectively (Figure 2A).

**Figure 3:**
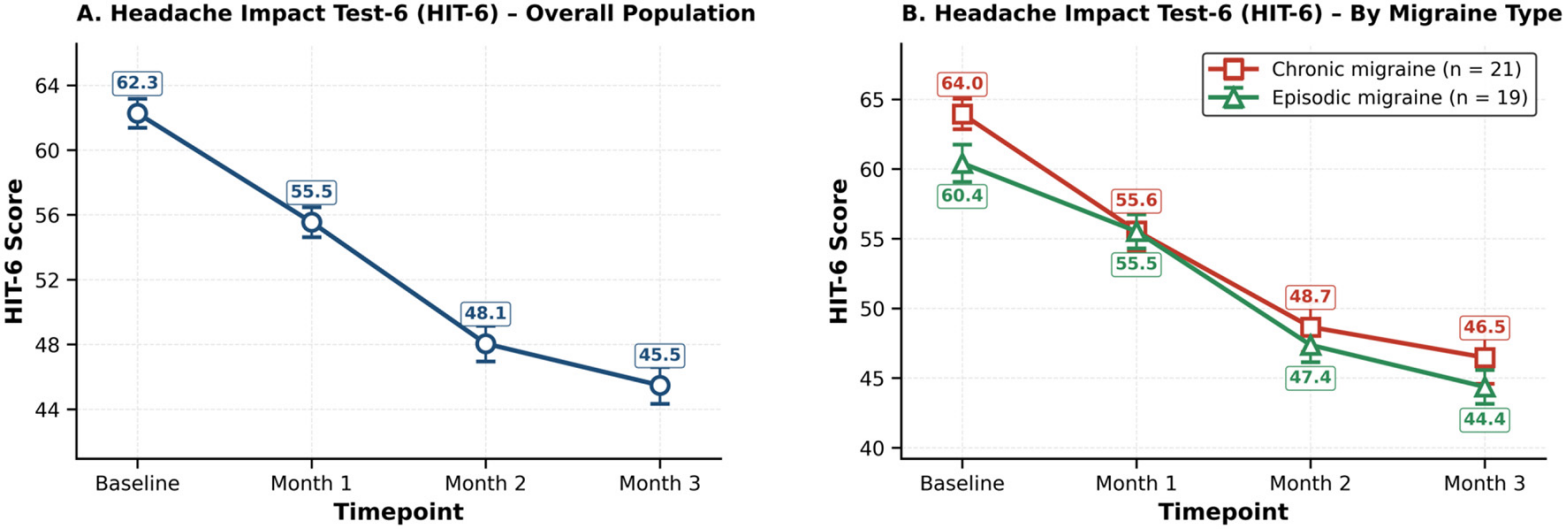
Headache impact test-6 (HIT-6). **(A)** HIT-6 overall population. **(B)** HIT-6 score by migraine type.

**Figure 4:**
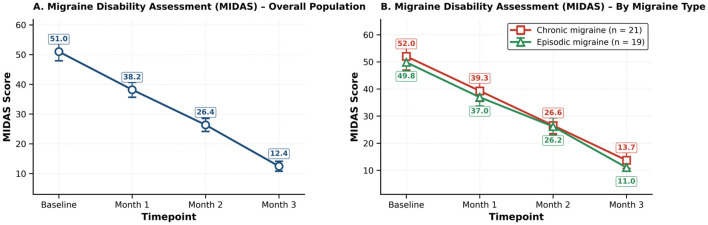
Migraine disability assessment (MIDAS). **(A)** MIDAS scores overall population. **(B)** MIDAS scores by migraine type.

**Figure 5:**
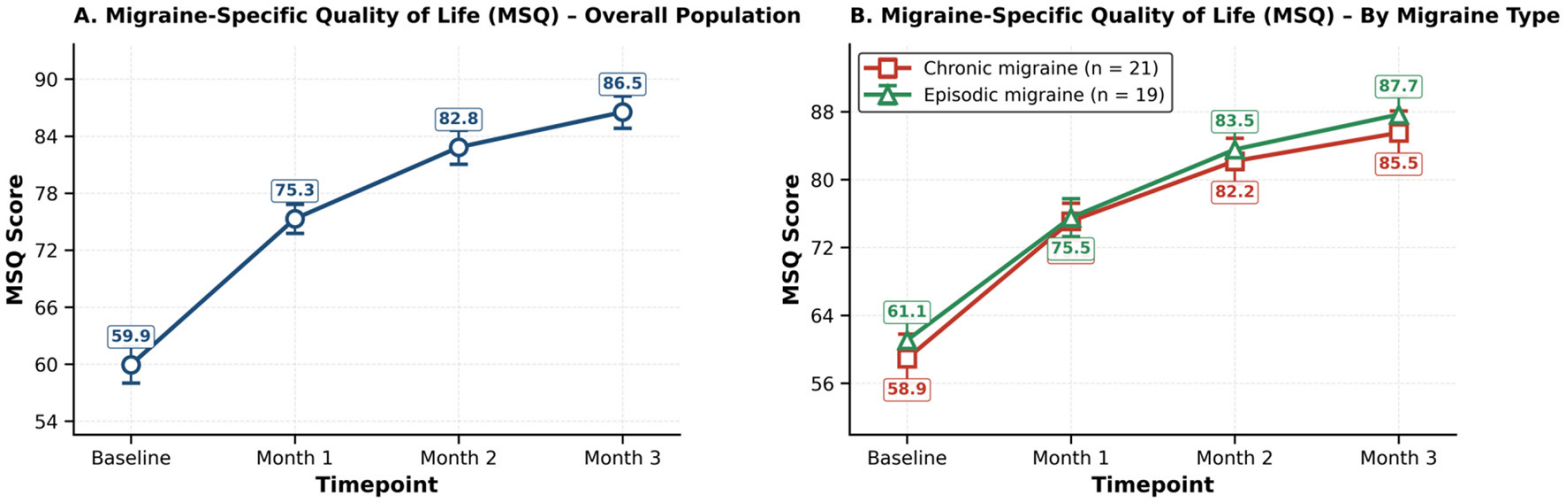
Migraine-specific quality of life questionnaire (MSQ). **(A)** MSQ scores overall population. **(B)** MSQ scores by migraine type.

Headache-related impact decreased over the same interval. The mean HIT-6 score declined from 62.27 ± 5.64 at baseline to 55.55 ± 5.83 at Month 1, 48.05 ± 6.95 at Month 2, and 45.47 ± 7.21 at Month 3, with a partial eta squared of 0.80 (Figure 3A). MIDAS scores showed a comparable downward trajectory, from 50.99 ± 19.24 at baseline to 38.18 ± 15.92, 26.36 ± 13.86, and 12.44 ± 10.58 at Months 1, 2, and 3, respectively (η^2^*p* = 0.83) (Figure 4A). Conversely, the MSQ score rose from 59.94 ± 12.21 at baseline to 75.30 ± 9.60 at Month 1, 82.82 ± 11.32 at Month 2, and 86.53 ± 10.71 at Month 3 (η^2^*p* = 0.79), reflecting an upward shift in migraine-specific quality of life across the follow-up period (Figure 5A).

### Treatment outcomes by migraine type

Stratified analyses were performed for chronic migraine (*n* = 21; Table 3A) and episodic migraine (*n* = 19; Table 3B). All five outcomes changed significantly across the four time points in both subgroups (all *P* = 0.001).

**Table 3A:** Treatment outcomes across chronic migraine patients follow-up intervals (*N* = 21).

Outcome	Baseline	Month 1	Month 2	Month 3	*P*-value
MMD (days)	23.19 ± 7.79	8.48 ± 6.71	4.19 ± 4.69	3.14 ± 3.85	0.001^**#**^
MHD (days)	24.29 ± 6.94	14.24 ± 8.36	8.71 ± 6.73	7.19 ± 4.61	0.001^**#**^
HIT-6	63.95 ± 5.00	55.57 ± 6.39	48.66 ± 8.24	46.47 ± 8.60	0.001^$^ (0.72)^@^
MIDAS	52.04 ± 23.22	39.30 ± 17.64	26.55 ± 13.93	13.71 ± 13.51	0.001^$^ (0.75)^@^
MSQ	58.91 ± 13.24	75.10 ± 9.72	82.17 ± 12.37	85.51 ± 11.83	0.001^$^ (0.68)^@^

Values are presented as mean ± standard deviation. MHD = monthly headache days; MMD = monthly migraine days; HIT-6 = Headache Impact Test-6; MIDAS = Migraine Disability Assessment; MSQ = Migraine-Specific Quality of Life Questionnaire. ^*^P-values calculated using Friedman test; ^$^*P*-values calculated using repeated measure ANOVA, *P* < 0.05 considered statistically significant, ^@^partial eta squared (η^2^*p*).

**Table 3B:** Treatment outcomes across episodic migraine patients follow-up intervals (*N* = 19).

Outcome	Baseline	Month 1	Month 2	Month 3	*P*-value
MMD (days)	10.73 ± 1.96	7.89 ± 1.79	5.26 ± 1.85	2.63 ± 1.34	0.001^**#**^
MHD (days)	13.37 ± 1.01	9.42 ± 1.83	6.84 ± 1.54	3.84 ± 1.30	0.001^**#**^
HIT-6	60.42 ± 5.85	55.52 ± 5.32	47.37 ± 8.00	44.37 ± 5.32	0.001^$^ (0.97) ^@^
MIDAS	49.84 ± 14.15	36.96 ± 14.15	26.15 ± 14.15	11.04 ± 5.97	0.001^$^ (0.94) ^@^
MSQ	61.07 ± 11.20	75.53 ± 9.72	83.54 ± 10.33	87.66 ± 9.51	0.001^$^ (0.98) ^@^

Values are presented as mean ± standard deviation. MHD = monthly headache days; MMD = monthly migraine days; HIT-6 = Headache Impact Test-6; MIDAS = Migraine Disability Assessment; MSQ = Migraine-Specific Quality of Life Questionnaire. ^*^P-values calculated using Friedman test; ^$^*P*-values calculated using repeated measure ANOVA, *P* < 0.05 considered statistically significant, ^@^Partial eta squared (η^2^*p*).

In the chronic migraine subgroup, MMD fell from 23.19 ± 7.79 at baseline to 8.48 ± 6.71 at Month 1, 4.19 ± 4.69 at Month 2, and 3.14 ± 3.85 at Month 3 (Figure 1B). MHD decreased in parallel, from 24.29 ± 6.94 at baseline to 14.24 ± 8.36, 8.71 ± 6.73, and 7.19 ± 4.61 at the three follow-up visits (Figure 2B). HIT-6 scores moved from 63.95 ± 5.00 to 46.47 ± 8.60 between baseline and Month 3 (η^2^*p* = 0.72; Figure 3B), MIDAS scores from 52.04 ± 23.22 to 13.71 ± 13.51 (η^2^*p* = 0.75; Figure 4B), and MSQ scores from 58.91 ± 13.24 to 85.51 ± 11.83 (η^2^*p* = 0.68; Figure 5B).

In the episodic migraine subgroup, the magnitude of change in MMD and MHD was smaller in absolute terms, consistent with the lower baseline values. MMD decreased from 10.73 ± 1.96 at baseline to 7.89 ± 1.79, 5.26 ± 1.85, and 2.63 ± 1.34 at Months 1, 2, and 3, respectively (Figure 1B), while MHD declined from 13.37 ± 1.01 to 9.42 ± 1.83, 6.84 ± 1.54, and 3.84 ± 1.30 over the same interval (Figure 2B). HIT-6 scores decreased from 60.42 ± 5.85 at baseline to 44.37 ± 5.32 at Month 3 (η^2^*p* = 0.97; Figure 3B); MIDAS scores fell from 49.84 ± 14.15 to 11.04 ± 5.97 (η^2^*p* = 0.94; Figure 4B); and MSQ scores increased from 61.07 ± 11.20 to 87.66 ± 9.51 (η^2^*p* = 0.98; Figure 5B).

Across all five outcomes, both subgroups followed the same direction of change observed in the overall population, with the steepest decline in headache frequency occurring between baseline and Month 1 and a more gradual change thereafter, as illustrated in panels B of Figures –. By Month 3, mean MMD (3.14 vs. 2.63), MMD (7.19 vs. 3.84), HIT-6 (46.47 vs. 44.37), MIDAS (13.71 vs. 11.04), and MSQ (85.51 vs. 87.66) values were comparable between the chronic and episodic subgroups, despite the substantial baseline differences in headache and migraine day counts (23.19 vs. 10.73 for MMD; 24.29 vs. 13.37 for MHD).

## Discussion

This three-month retrospective cohort study examined every-other-day rimegepant as preventive therapy in 40 patients with chronic or episodic migraine who had previously shown an inadequate response to standard preventive treatment. Across all five outcome measures, monthly migraine days (MMD), monthly headache days (MHD), HIT-6, MIDAS, and MSQ, significant change from baseline was observed at every follow-up visit, with the steepest shift occurring between baseline and month 1 and a more gradual change thereafter (all *P* = 0.001; partial η^2^ values for the patient-reported outcomes ranging from 0.68 to 0.98). Reductions were evident in both migraine subtypes, and by month 3 the chronic and episodic groups had converged to broadly similar mean values despite their substantial baseline differences in headache and migraine day counts.

These observations sit within a treatment landscape in mainland China that remains relatively narrow for migraine prevention. Flunarizine and other traditional agents continue to dominate routine prescribing, and among targeted therapies only the CGRP monoclonal antibody erenumab has secured formal regulatory approval for prevention; rimegepant is currently approved by the National Medical Products, Administration only for acute treatment, so its use as a preventive agent in this cohort, including in chronic migraine, was off-label (33–35). This regulatory context is relevant when interpreting both the patient population and the external applicability of the present findings.

The direction and magnitude of the changes we observed are broadly consistent with the pivotal phase 2/3 randomized trial of rimegepant, which showed that 75 mg every other day reduced MMD from baseline more than placebo over 12 weeks (20), and with the open-label long-term safety study (BHV3000-201), in which sustained reductions in MMD and improvements in health-related quality of life were maintained over 52 weeks (36). Our cohort showed numerically larger reductions in MMD than those reported in the controlled-trial setting, which most likely reflects differences in study design and population, particularly the inclusion of treatment-experienced patients with high baseline frequency rather than treatment-naive participants enrolled into a randomized trial, rather than greater pharmacological effect. Network meta-analyses of phase 3 trials have consistently shown comparable effect sizes across CGRP-targeted preventive therapies regardless of molecule class (37, 38), and a head-to-head phase 4 trial of galcanezumab vs. rimegepant in episodic migraine found no superiority on the primary ≥50%-responder endpoint (62% vs. 61%) (39). Our results align with the broader pattern these studies describe, although the absence of a comparator arm precludes any direct claim of equivalence.

Improvements in patient-reported outcomes mirrored the change in headache frequency. The HIT-6 score declined by approximately 17 points overall, moving group means from the “severe impact” range at baseline (62.27) into the “little or no impact” category at month 3 (45.47), based on the validated cut-points of the instrument (30). MIDAS scores shifted from values consistent with severe disability (Grade IV) into the lower disability range, and MSQ improved by roughly 27 points overall, a change that exceeds the within-patient minimum important differences of 7.5, 10.9 points originally established for this instrument (40). Patient-reported outcomes are increasingly viewed as central to assessing treatment benefit in migraine, since they capture functional and quality-of-life dimensions not reflected in headache day counts alone (41). The convergent improvement we observed across HIT-6, MIDAS, and MSQ supports the view that the reductions in migraine and headache days translated into meaningful gains in daily functioning rather than statistical change alone.

Both subgroups followed similar trajectories. In chronic migraine, MMD fell from 23.19 at baseline to 3.14 at month 3, while in episodic migraine it fell from 10.73 to 2.63, with comparable proportional changes in MHD, HIT-6, MIDAS, and MSQ. This pattern is consistent with what would be expected of an agent acting on a shared CGRP-mediated trigeminovascular pathway (42), and with previous network meta-analyses suggesting that effect sizes for CGRP-targeted preventives are similar across episodic and chronic populations (37, 38). The observation that both groups achieved broadly comparable end-of-study values argues against restricting use of preventive rimegepant to a particular frequency phenotype, although the small subgroup sizes limit the strength of any inference.

The within-patient trajectory, large early change followed by progressive but smaller monthly gains, is in line with reports that the onset of benefit with CGRP-targeted therapies can occur within the first weeks of exposure but that maximal response often accrues over subsequent months (43, 44). This temporal pattern is clinically important because patients and clinicians sometimes judge a preventive trial too early, which patient-preference data suggest is one of the determinants of perceived treatment value (45). The oral, every-other-day regimen of rimegepant offers a practical profile distinct from monthly or quarterly subcutaneous monoclonal antibodies, with the additional feature of dual labeling for acute and preventive use in some jurisdictions, although the latter does not currently apply in mainland China (34, 35).

In line with available records, no patient discontinued treatment because of an adverse event, and no serious adverse events were documented. Reported side effects included nausea, general discomfort, drowsiness, tension-type headache, and motion sickness, but these were not collected with a standardized instrument and frequencies, severity grades, and onset timings cannot be reported with confidence. Hepatic safety was not monitored in this cohort. Available data from controlled trials and post-marketing summaries suggest that clinically significant transaminase elevations are uncommon and that no confirmed cases of clinically apparent rimegepant-induced liver injury have been described to date (46), but routine hepatic monitoring should still be considered in future prospective work in Chinese populations to extend that safety database.

Real-world evidence on rimegepant remains limited in scope, particularly outside Western settings. A recent Chinese prospective multicentre study of patient-reported outcomes (27), the Italian GAINER study (47), and the Danish nationwide register study (48) together suggest that effectiveness and tolerability profiles in routine practice broadly track those reported in randomized trials, although the Danish data also highlighted that 45% of initiators redeemed only a single prescription, pointing to substantial early discontinuation in unselected populations. The International Headache Society has recently published guidelines specifically for the conduct of real-world evidence studies in migraine and cluster headache (49), and future work in this area, including ours, would benefit from prospective alignment with those recommendations.

### Strengths and limitations

The principal strengths of this study are the use of a standardized set of validated outcome measures (MMD, MHD, HIT-6, MIDAS, MSQ), longitudinal follow-up at three predefined post-baseline visits, and a population that reflects routine clinical practice in a setting where preventive options are limited.

Several limitations must temper interpretation. First, this is a single-center, single-arm retrospective cohort with no control group, no placebo arm, and no active comparator. Causal attribution of the observed changes to rimegepant is therefore not possible. Migraine outcomes are particularly susceptible to regression to the mean because patients typically present and enroll during periods of atypically high headache frequency, with some symptom improvement expected on repeat measurement irrespective of treatment (50). Placebo response rates in migraine prevention trials commonly fall in the 20%−30% range and have been shown to drift upward over recent decades (51, 52), and contextual effects from regular structured follow-up may themselves contribute to apparent benefit (53). Taken together, these factors mean the present associations should be interpreted as hypothesis-generating rather than as evidence of pharmacological efficacy.

Second, the sample size is small (*N* = 40) and the chronic and episodic subgroups (*n* = 21 and *n* = 19) are underpowered for between-group inferential comparison. Third, the three-month observation window is too short to characterize the durability of response, the timing of any plateau or attenuation, or longer-term adherence patterns. Fourth, seasonal and chronobiological variability in attack frequency was not systematically recorded; given the well-documented temporal patterning of migraine (54), some component of the within-patient change may reflect environmental rather than pharmacological influences. Fifth, only patients with complete medical records across the full three-month period were eligible, which introduces a survivorship bias likely to enrich the analysis with patients tolerating and continuing treatment, an effect amplified by the discontinuation rates reported in real-world CGRP-targeted-therapy cohorts (48, 55). Sixth, adverse events were not collected through a standardized instrument, precluding a comprehensive safety characterization. Finally, the high acquisition cost of rimegepant and its current off-label status for prevention in mainland China are practical and regulatory factors that further constrain the generalizability of these observations (56).

## Conclusion

In a treatment-experienced cohort of patients with chronic or episodic migraine, every-other-day rimegepant was associated with progressive reductions in migraine and headache day counts and with parallel improvements in headache-related impact, disability, and migraine-specific quality of life over 3 months. Both subgroups showed concordant changes, and tolerability appeared acceptable within the limits of the available records. Given the retrospective, single-center, uncontrolled design, these results should be regarded as hypothesis-generating. Prospective, multicenter, controlled studies with longer follow-up, standardized adverse-event capture, and pre-specified analyses across migraine subtypes are needed to establish causal effectiveness, define durability of response, identify clinical or biomarker predictors of response, and inform the optimal positioning of rimegepant within preventive treatment algorithms, particularly in the Chinese setting, where high-quality long-term prevention data with this molecule remain limited.

## Data Availability

The raw data supporting the conclusions of this article will be made available by the authors, without undue reservation.
